# High co‐expression of SLC7A11 and GPX4 as a predictor of platinum resistance and poor prognosis in patients with epithelial ovarian cancer

**DOI:** 10.1111/1471-0528.17327

**Published:** 2022-12-09

**Authors:** Xiaodong Wu, Shizhen Shen, Jiale Qin, Weidong Fei, Fengyun Fan, Jiaxin Gu, Tao Shen, Tao Zhang, Xiaodong Cheng

**Affiliations:** ^1^ Department of Gynaecologic Oncology Women's Hospital, Zhejiang University School of Medicine Hangzhou China; ^2^ Department of Ultrasound Women's Hospital, Zhejiang University School of Medicine Hangzhou China; ^3^ Department of Pharmaceutics Women's Hospital, Zhejiang University School of Medicine Hangzhou China; ^4^ Zhejiang Provincial Key Laboratory of Precision Diagnosis and Therapy for Major Gynecological Diseases Women's Hospital, Zhejiang University School of Medicine Hangzhou, Zhejiang China

**Keywords:** ferroptosis, GPX4, ovarian cancer, platinum resistance, poor prognosis, SLC7A11

## Abstract

**Objective:**

The aim was to assess the expression levels of SLC7A11 and GPX4 in relation to platinum resistance and prognosis in patients with epithelial ovarian cancer (EOC).

**Design:**

A retrospective cohort study.

**Setting:**

Women's Hospital, Zhejiang University School of Medicine, Hangzhou, China.

**Population or Sample:**

We included 192 eligible patients from hospital between January 2002 and December 2018.

**Methods:**

We retrospectively analysed the medical records of patients with EOC. Surgical specimens of EOC were stained for SLC7A11 and GPX4. Survival analysis was performed using the Kaplan–Meier and Cox regression methods.

**Main Outcome Measures:**

Clinical end points include platinum‐free interval (PFI), progression‐free survival (PFS) and overall survival (OS).

**Results:**

Patients with high co‐expression levels of SLC7A11 and GPX4 had a 60‐fold higher risk of platinum resistance compared with those with low co‐expression (risk ratio, 60.46; 95% confidence interval [CI] 22.76–160.58; *p* < 0.001). Moreover, high co‐expression level of SLC7A11 and GPX4 was an independent prognostic factor for poor OS (*p* < 0.001, hazard ratio [HR] 4.44, 95% CI, 2.77–7.14) and poor PFS (*p* < 0.001, HR = 5.73, 95% CI, 3.86–8.73). For in vitro experiments, SLC7A11 and GPX4 expression were both upregulated in platinum‐resistant cells compared with their parental ovarian cancer cells, and siRNA‐induced SLC7A11 and GPX4 inhibition decreased platinum resistance.

**Conclusions:**

High expression levels of SLC7A11 and GPX4 are associated with platinum resistance in EOC patients. High co‐expression of SLC7A11 and GPX4 may be a significant independent prognostic factor and a potential therapeutic target for platinum resistance in EOC patients.

## 1 INTRODUCTION

Ovarian cancer is the most lethal gynaecological malignancy, with a 5‐year survival rate of 30–50%.^1^ Epithelial ovarian cancer (EOC) accounts for >95% of ovarian malignancies.^2^ Most patients with ovarian cancer are diagnosed at an advanced stage (International Federation of Gynaecology and Obstetrics [FIGO] 2000 Stage III/IV), and over 75% of patients with late‐stage ovarian cancer die of the disease. Although the development of targeted therapies has gradually changed the prognosis of patients with platinum‐sensitive EOC,^3^, ^4^, ^5^, ^6^ effective treatments for patients with platinum‐resistant EOC are still lacking.^7^, ^8^ Therefore, chemotherapy is still the best therapeutic approach. There is an urgent need to improve patient stratification for therapeutic strategies to overcome treatment resistance and search for new therapeutic targets for platinum resistance in patients with ovarian cancer.

Ferroptosis is a cell death modality that differs from other forms of cell death morphologically, biochemically and genetically, and is characterised by an accumulation of iron‐dependent lethal lipid reactive oxygen species (ROS).^9^ Glutathione peroxidase 4 (GPX4) is the only peroxidase in mammals that can reduce phospholipid hydroperoxides within cell membranes, and it has been determined to be a central regulator of ferroptosis.^10^ Therefore, GPX4 activity is considered a ferroptosis molecular marker.^11^

Stockwell et al.^12^ discovered that the cystine/glutamate antiporter SLC7A11 (also known as xCT) could import cystine, promote glutathione (GSH) biosynthesis, and subsequently facilitate the GPX4‐mediated detoxification of lipid peroxides to inhibit ferroptosis. SLC7A11 plays a significant role in regulating GPX4 activity and protecting cells from ferroptosis.^9^, ^13^ One of the main classes of ferroptosis inducers (such as erastin) is a promising anticancer strategy that works by inhibiting SLC7A11.^14^ Therefore, SLC7A11 may be a potential therapeutic target in cancer by inducing ferroptosis. Moreover, recent studies have shown that ovarian cancers with wild‐type *BRCA* are sensitive to targeted‐SLC7A11 ferroptosis inducers.^15^ It is well known that *BRCA* wild‐type ovarian cancer is resistant to DNA damage and poly(ADP‐ribose) polymerases (PARP) inhibitors. Therefore, a link may exist between high expression levels of SLC7A11, ferroptosis and platinum resistance in ovarian cancer. Ferroptosis may also be a new therapeutic target in patients with platinum‐resistant ovarian cancer.

However, the link between SLC7A11 and GPX4 expression in platinum‐resistant ovarian cancer and its association with patient prognosis has not yet been reported. In this study, we aimed to verify the association between the co‐expression levels of SLC7A11 and GPX4 with platinum resistance and poor prognosis in patients with EOC.

## 2 MATERIALS AND METHODS

### 2.1 Study design and patient characteristics

This retrospective cohort study included patients with platinum‐resistant EOC at the Women's Hospital, Zhejiang University School of Medicine, from January 2002 to December 2018. Patients were eligible if they met the following criteria:

- histological diagnosis of EOC;
- having undergone ovarian cancer staging or cytoreductive surgery at our hospital;
- having received the taxane/platinum (TP) protocol set up by our hospital after primary surgery;
- having completed the first course of TP chemotherapy at our hospital and having had an uneventful completion of all courses of the TP regimen;
- a follow‐up of at least 12 months after the last course of chemotherapy.

The exclusion criteria were as follows:

- pregnant or breastfeeding;
- undertaking a non‐TP chemotherapy regimen;
- treatment regimen changed due to TP‐related side effects (such as serositis or allergy);
- undergoing neoadjuvant chemotherapy before primary surgery.

The flow diagram of patient inclusion and exclusion is illustrated in Figure S1.

Platinum‐free interval (PFI) was defined as the time between the end of the last platinum‐based chemotherapy and disease progression. Using a PFI of 6 months as the threshold, the patients were divided into the platinum‐resistant (<6 months) and platinum‐sensitive (≥6 months) groups.^10^ Follow‐up was performed by outpatient review or telephone consultation. For patients who did not complete all TP courses at our hospital, we utilised their follow‐up information to ensure that they have received all recommended courses at other hospitals and obtained the specific date of their last chemotherapy (or if they did not receive it). Finally, 70 platinum‐resistant patients (group 1) were included in our study (Figure S1).

After the platinum‐resistant group was selected, we established a platinum‐sensitive group as the control, matched by FIGO stage, age and tumour grade, in a ratio of 1:3. We employed a random sampling method to select an equal number of patients who experienced recurrence between 6 and 12 months (group 2), more than 12 months but not more than 60 months (group 3), or no recurrence for at least five natural years (group 4, including patients who experienced recurrence with a PFI more than 60 months, and patients who had no recurrence until at least the fifth postoperative year to the last follow‐up) after the end of the last platinum‐based chemotherapy for the platinum‐sensitive group.

The following clinical information was recorded for each subject: age, International Federation of Gynaecology and Obstetrics (FIGO) stage, tumour grade, preoperative serum CA125 level, ascitic fluid volume, date of the initial surgical procedure, classification of the surgery, and date of completion of TP chemotherapy. Surgery was defined as optimal cytoreduction (residual lesion <1.0 cm) or suboptimal cytoreduction (residual lesion >1.0 cm) according to the GOG‐172 criteria.^16^ Surgery was classified as stage I–IV according to the FIGO 2014 guidelines.^17^ The methods for calculating progression‐free survival (PFS) and overall survival (OS) were performed as described in a previous study.^18^

### 2.2 Immunohistochemistry

Formalin‐fixed and paraffin‐embedded (FFPE) tissue samples obtained from eligible patients with EOC were used for immunohistochemical analyses. FFPE sections (5‐μm thickness) were deparaffinised in xylene, rehydrated in a graded ethanol series, and quenched with endogenous peroxides. The sections were then incubated with anti‐SLC7A11 (1:600 in NCM universal antibody diluent, ab37185; Abcam) and anti‐GPX4 antibodies (1:300 in NCM universal antibody diluent, ab125066; Abcam). Two serial sections from the same paraffin‐embedded block were stained with the anti‐SLC7A11 and anti‐GPX4 antibodies. Staining was visualised using a GTVision Detection System (GTVision, GK6007710A; Gene Tech). The sections were counterstained with haematoxylin, dehydrated and mounted. The negative control was processed without a primary antibody, and only tumour samples containing at least 70% tumour tissue were included, as described previously. The percentage of SLC7A11 and GPX4 expression was calculated semiquantitatively using positive cells in an entire region containing ovarian cancer cells in each section. The percentage of cells with positive staining was scored using a scale from 1 to 4, wherein 1 indicates 0–25% of cells were positively stained, 2 indicates 26–50%, 3 indicates 51–75%, and 4 indicates 76–100%. The staining intensities were scored from 0 to 3, where 0 = negative, 1 = weak, 2 = moderate and 3 = strong. Subsequently, the percentages and intensity scores were multiplied to obtain a total score ranging from 0 to 12.^19^ Scores of 8–12 were defined as high expression and scores of 0–7 as low expression.^20^ All pathological diagnoses and staining results were confirmed by two expert pathologists who were blinded to the clinical details of the cases. This study was approved by the Ethics Committee of the Women's Hospital, Zhejiang University School of Medicine (November 2018, IRB‐20200227‐R). Written informed consent was exempted by the Institutional Review Board.

### 2.3 Experimental protocol of the in vitro cell study

For in vitro cytological experiments, we used the following methods: cell culture, cell viability assay, western blot analysis, RNA extraction, RT‐qPCR analysis and siRNA transfection. All detailed protocols of the cytological experiments and GEO database analysis are presented in Tables S1 and S2.

### 2.4 Statistical analyses

Statistical analyses were conducted using an SPSS (version 18.0) statistical software package (IBM Corp.). The Kolmogorov–Smirnov test was used to test for normality in continuous data. Student's *t*‐test and the Mann–Whitney *U*‐test were used to compare normally and non‐normally distributed data, respectively. The Chi‐square test was used for comparisons between groups, and partitions of the Chi‐square method were used for pairwise comparisons. In cases where the number of subjects was less than five, Fisher's exact test was used for the contingency tables, and the remaining data were tested using the Chi‐square test. A logistic regression model was also applied to sort risk factors based on their strengths. Correlation analysis was performed using Spearman's correlation coefficient (two‐tailed) and scatter plot analyses. We used the Kaplan–Meier method to calculate the OS curves and the log‐rank test to assess the differences in the survival rates. Subsequently, univariate and multivariate Cox analyses were conducted, involving all potential predictive factors. Receiver operating characteristic (ROC) curves were used to discriminate the potential of each important factor identified in the above‐described comparative analysis. When the area under the curve (AUC) had a low 95% confidence interval (CI) (>0.5), discrimination was considered significant. Statistical significance was set at *p*‐values <0.05 (two‐tailed).

## 3 RESULTS

Initially, 280 patients were selected for this study. After excluding 39 patients whose FFPE tumour tissue content was <70% and 49 patients whose staining results were unsatisfactory, 192 patients were included in the final analysis. Figure S2 illustrates the inclusion of the participants in this study. After exclusion, there were no significant differences in FIGO stage, age or tumour grade among the four groups (*p* = 0.108). The median age of the included patients was 52 years (range, 31–72 years). Histologically, well‐differentiated tumours were observed in three patients (1.56%, 3/192), moderately differentiated tumours in 13 patients (6.77%, 13/192) and poorly differentiated tumours in the remaining 176 patients (91.67%, 176/192). Most patients (76.56%, 147/192) had FIGO stages III or IV, and the remaining 45 (23.43%, 45/192) had FIGO stages I or II. The optimal initial surgery was achieved in 79.17% (152/192) of the included patients.

When evaluating the differential expression of SLC7A11 and GPX4 in the four groups, only group 1 differed significantly from the other groups (Tables S3–S6). As such, we combined groups 2, 3 and 4 into a platinum‐sensitive group (*n* = 144), renamed group 1 the platinum‐resistant group (*n* = 48), and performed several analyses on these two groups. Overall, there were 38 deaths (79.17%, 38/48) in the platinum‐resistant group and 53 deaths (36.81%, 53/144) in the platinum‐sensitive group (*p* < 0.001). Among the 53 deaths, one patient died of breast cancer at 138 months post‐operation without recurrence of ovarian cancer.

As we failed to identify the exact date of death in three patients, 189 patients were eligible for OS calculation. The median OS was 56 months (range, 6–209 months). Similarly, only 189 patients were eligible for PFS calculation, and the median PFS was 18 months (range, 2–209 months). As we failed to identify the exact date of recurrence in three patients and 44 patients had no recurrence, only 145 patients were eligible for calculation of the PFI.

### 3.1 Evaluation of SLC7A11 and GPX4 expression in ovarian cancer

**FIGURE 1:**
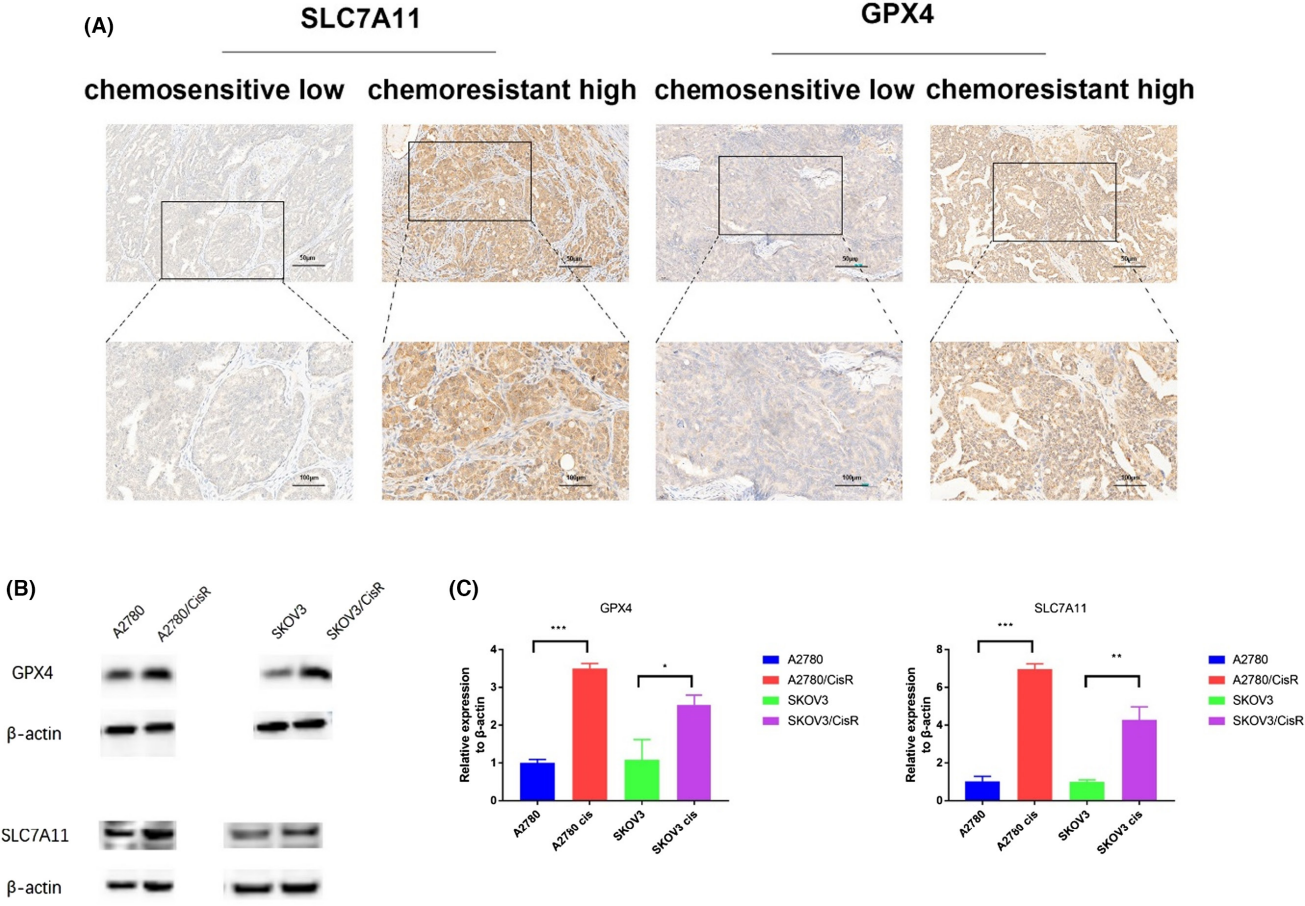
Assessment of SLC7A11 and GPX4 expression in ovarian cancer tissues. (A) Representative micrographs of SLC7A11 and GPX4 staining from platinum‐resistant and platinum‐sensitive tissues from tissues of patients with ovarian cancer. Scale bars (50 and 100 micrometers) are shown on the lower right. (B) SLC7A11 and GPX4 expression were both upregulated in A2780/CisR and SKOV3/CisR cells compared with A2780 and SKOV3 cells, respectively, as revealed via western blotting. (C) Quantitative PCR analysis showed that SLC7A11 and GPX4 transcription were both upregulated in A2780/CisR and SKOV3/CisR cells. *p < 0.05; **p < 0.01; ***p < 0.001.

Staining for SLC7A11 and GPX4 was observed in the cytoplasm of EOC tissues obtained from the included patients (Figure 1A). EOC cases were classified as SLC7A11‐low expression or SLC7A11‐high expression and GPX4‐low expression or GPX4‐high expression. Of the 192 included patients, 43 (22.40%, 43/192) had SLC7A11‐high expression and 44 (22.92%, 44/192) had GPX4‐high expression.

SLC7A11 and GPX4 were then selected for validation using western blotting and RT‐qPCR analyses in two cell lines (human ovarian cancer cells A2780, SKOV3 and their respective platinum‐resistant cells subline A2780/CisR, SKOV3/CisR) to confirm further the alterations in protein expression revealed by immunohistochemical analyses. SLC7A11 and GPX4 expression were both upregulated in platinum‐resistant cell sublines compared with their parental ovarian cancer cell lines, as determined via western blotting (Figure 1B). Quantitative PCR analysis showed that SLC7A11 and GPX4 transcription were both upregulated in platinum‐resistant cell sublines compared with the parental ovarian cancer cell lines (Figure 1C). These changes were consistent with the immunohistochemical results in EOC tissues. Conversely, the inhibition of SLC7A11 and GPX4 decreased platinum resistance in ovarian cancer cells (Figure S3).

### 3.2 The association of SLC7A11 and GPX4 expression with clinicopathological parameters

High SLC7A11 protein expression was significantly associated with FIGO stage III/IV (*p* = 0.038) and suboptimal initial surgery (*p* = 0.001) (Table 1). On the other hand, a high expression of GPX4 was significantly associated with a higher level of serum CA125 (*p* = 0.010), higher ascitic fluid volume (*p* = 0.002) and suboptimal initial surgery (*p* = 0.001) (Table 2). As expected, the proportions of cases with a high expression of SLC7A11 and GPX4 were 62.50% (30/48 cases) and 68.75% (33/48 cases), respectively, in platinum‐resistant ovarian cancer tissues and only 9.03% (13/144 cases) and 7.64% (11/144 cases), respectively, in platinum‐sensitive tissues, both of which were significant (both *p* < 0.001) (Tables 1 and 2).

**TABLE 1:** Association of SLC7A11 expression with clinicopathological parameters

Variable	SLC7A11‐low expression, *n* (%)	SLC7A11‐high expression, *n* (%)	*p*‐value
Number of patients age, years	149 (77.60)	43 (22.40)	0.316
<50	50 (73.53)	18 (26.47)	
≥50	99 (79.84)	25 (20.16)	
FIGO stage			
I/II	40 (88.89)	5 (11.11)	0.038*
III/IV	109 (74.15)	38 (25.85)	
Tumour grade			
I/II	13 (81.25)	3 (18.75)	1.00^a^
III	136 (77.27)	40 (22.73)	
Ascitic fluid volume			
<500 ml	96 (82.05)	21 (17.95)	0.065
≥500 ml	53 (70.67)	22 (29.33)	
Serum CA125			
<500 U/ml	76 (80.85)	18 (19.15)	0.291
≥500 U/ml	73 (74.49)	25 (25.51)	
Primary surgery			
Optimal	126 (82.89)	26 (17.11)	0.001**
Suboptimal	23 (57.50)	17 (42.50)	
Platinum resistance			
Sensitive	131 (90.97)	13 (9.03)	<0.001***
Resistant	18 (37.50)	30 (62.50)	

Abbreviation: FIGO, International Federation of Gynaecology and Obstetrics.

*Note*: Categorical data are presented as absolute values (%). *p*‐values were calculated using the Chi‐square test.

^a^ These values were calculated using Fisher's exact tests, as more than 20% of the cells had expected frequencies <5.

*p < 0.05; **p < 0.01; ***p < 0.001.

**TABLE 2:** Relation between GPX4 expression and clinicopathological parameters

Variable	GPX4‐low expression *n* (%)	GPX4‐high expression *n* (%)	*p*‐value
Number of patients age, years	148 (77.08)	44 (22.92)	
<50	51 (75.00)	17 (25.00)	0.611
≥50	97 (78.23)	27 (21.77)	
FIGO stage			
I/II	38 (84.44)	7 (15.56)	0.179
III/IV	110 (74.83)	37 (25.17)	
Tumour grade			
I/II	13 (81.25)	3 (18.75)	1.00^a^
III	135 (76.70)	41 (23.30)	
Ascitic fluid volume			
<500 ml	99 (84.62)	18 (15.38)	0.002**
≥500 ml	49 (65.33)	26 (34.67)	
Serum CA125			
<500 U/ml	80 (85.11)	14 (14.89)	0.010*
≥500 U/ml	68 (69.39)	30 (30.61)	
Primary surgery			
Optimal	125 (82.24)	27 (17.76)	0.001**
Suboptimal	23 (57.50)	17 (42.50)	
Platinum resistance			
Sensitive	133 (92.36)	11 (7.64)	<0.001***
Resistant	15 (31.25)	33 (68.75)	

*Note*: Categorical data are presented as absolute values (%). *p*‐values were calculated using the Chi‐square test.

Abbreviation: FIGO, International Federation of Gynaecology and Obstetrics.

^a^ These values were calculated using Fisher's exact tests, as more than 20% of the cells had expected frequencies <5.

*p < 0.05; **p < 0.01; ***p < 0.001.

Moreover, there was a significant positive correlation between SLC7A11 and GPX4 expression in EOC tissues of our included 192 patients, as shown in Figure S4A (*r* = 0.677, Pearson correlation). Specifically, the percentage of SLC7A11 high‐expression ovarian cancer tissues with a high GPX4 expression was 60.47% (26/43 cases), whereas it was only 12.08% (18/149 cases) in SLC7A11 low‐expression tissues; this difference was statistically significant (*p* < 0.001). Similar results were obtained using the GSE66957 dataset, downloaded from the GEO database (Figure S4B).

### 3.3 High protein expression of SLC7A11 and GPX4 was associated with platinum resistance

The scatter diagram (Figure S5A) shows that a higher immunohistochemical score for SLC7A11 was associated with a shorter PFI. The median SLC7A11 immunohistochemical score in platinum‐resistant cases was higher than in platinum‐sensitive cases (8 vs. 4, *p* < 0.001). Similarly, the median GPX4 immunohistochemical score was higher in platinum‐resistant cases than in platinum‐sensitive cases (9 vs. 3, *p* < 0.001, Figure S5B).

To improve the predictive efficacy of platinum resistance in ovarian cancer, a combination of both protein expression of SLC7A11 and GPX4 was explored. Logistic regression was used to estimate the odds ratio (OR) and the results revealed that SLC7A11 and GPX4 had significantly positive correlations with platinum resistance (OR = 1.588 and 1.931, respectively; Table S7). Thus, the total score (referred to as protein expression) representing the sum of the immunohistochemical scores of 1.588*SLC7A11 and 1.931*GPX4 (SLC7A11‐GPX4) in the same patient was calculated according to the logistic regression model. Figure S5C shows that a higher SLC7A11‐GPX4 score was associated with a shorter PFI.

Receiver operating characteristic curve analysis showed an AUC value of 0.947 (95% CI 0.917–0.977), indicating its discriminatory potential for platinum resistance in EOC patients (Figure S3D). The optimal SLC7A11‐GPX4 score cut‐off value for platinum resistance was 22.935, with 83.33% sensitivity and 92.36% specificity. Patients with SLC7A11‐GPX4 scores ≥23 had a 60‐fold higher risk of platinum resistance than those with SLC7A11‐GPX4 scores <23 (risk ratio = 60.455, 95% CI 22.760–160.579, *p* < 0.001). Thus, an SLC7A11‐GPX4 score ≥23 was defined as a ‘high co‐expression level of SLC7A11‐GPX4’ and a score < 23 was defined as a ‘low co‐expression level of SLC7A11‐GPX4’ in subsequent analyses. In the present study, 78.431% (40/51) of cases with high co‐expression levels of SLC7A11‐GPX4 suffered from platinum resistance. In contrast, only 5.674% (8/141) of cases with a low co‐expression of SLC7A11‐GPX4 suffered from platinum resistance (*p* < 0.001).

### 3.4 High expression of SLC7A11 and GPX4 proteins was associated with poorer patient prognosis

**FIGURE 2:**
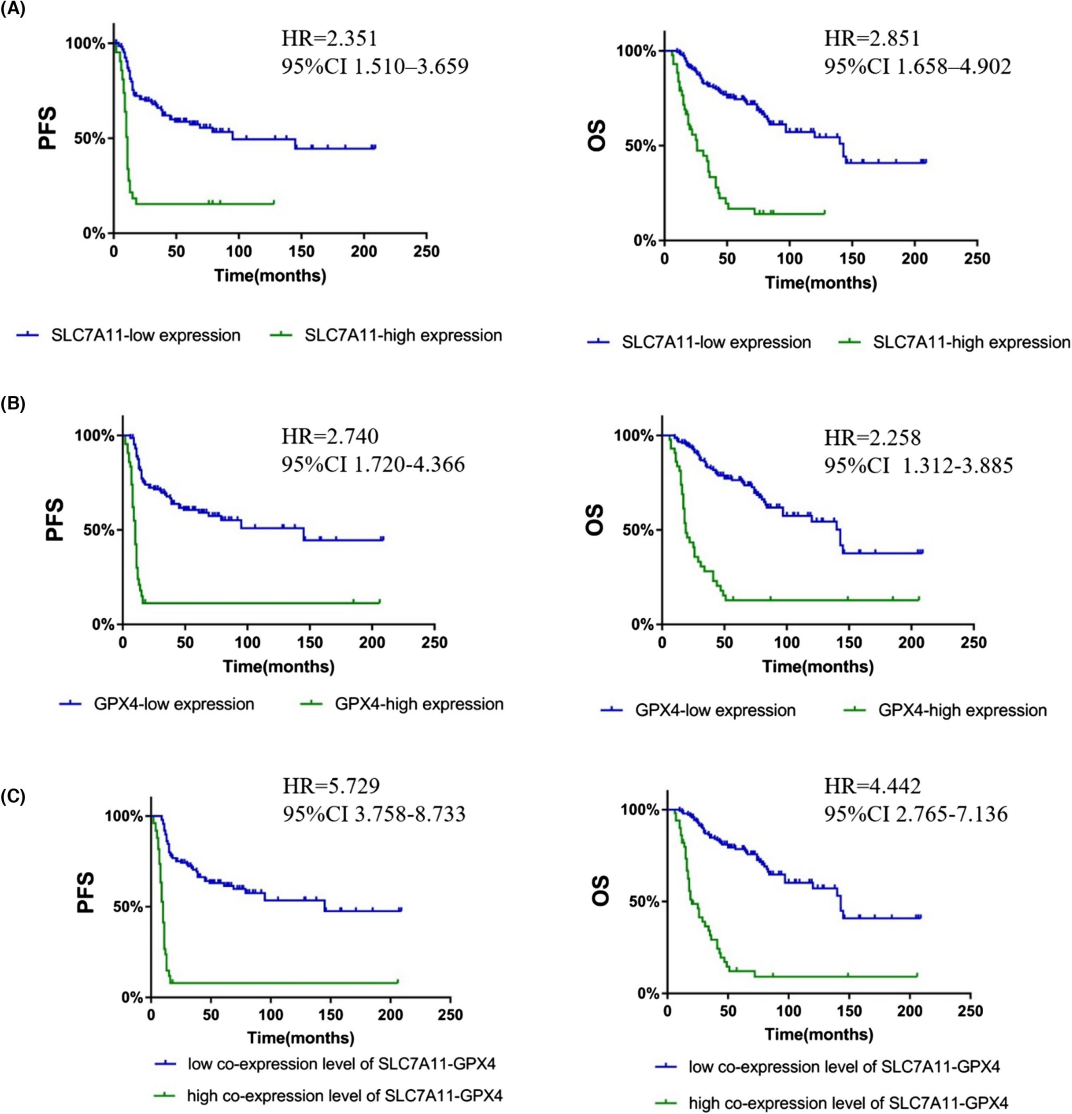
Analysis of the correlation between SLC7A11 and GPX4 expression and patient prognosis. (A) Kaplan–Meier survival curves for PFS and OS in ovarian cancer patients with different SLC7A11 expression levels. (B) Kaplan–Meier survival curves for PFS and OS in ovarian cancer patients with different GPX4 expression levels. (C) Analysis of SLC7A11‐GPX4 expression in the prognostic value of patients with ovarian cancer using Kaplan–Meier survival curves.

Kaplan–Meier survival curves (Figure 2A) showed that patients with ovarian cancer with a high SLC7A11 expression had significantly poorer PFS (*p* < 0.001) and OS (*p* < 0.001) than patients with a low expression of SLC7A11, which was confirmed by our univariate Cox (Table S8) and multivariate Cox proportional hazards regression analyses (hazard ratio [HR] = 2.351, 95% CI 1.510–3.659 for PFS, Table S9; HR 2.851, 95% CI 1.658–4.902 for OS, Table S10). The association between GPX4 expression and survival was similar to that observed for SLC7A11 (Figure 2B). Patients with a high co‐expression level of SLC7A11‐GPX4 had the shortest survival among EOC patients (Figure 2C). In contrast, multivariate Cox proportional hazards regression analysis revealed that a high co‐expression level of SLC7A11‐GPX4 was an independent prognostic factor for both PFS (*p* < 0.001, HR = 5.729, 95% CI 3.758–8.733, Table S11) and OS (*p* < 0.001, HR = 4.442, 95% CI 2.765–7.136, Table S12). Thus, our results suggest that a high co‐expression level of SLC7A11‐GPX4 was a superior predictor for poor prognosis and platinum resistance in ovarian cancer compared with the corresponding individual parameters (SLC7A11 or GPX4 expression).

## 4 DISCUSSION

### 4.1 Main findings

Although ovarian cancer treatment is a global problem, new therapeutic strategies are required for this disease. Increasing evidence has indicated that ferroptosis plays a crucial role in tumorigenesis and cancer therapy.^21^, ^22^ However, there are no robust predictive biomarkers of ferroptosis sensitivity for selecting tumours most likely to resist platinum‐based chemotherapy.^23^ In this study, we evaluated the predictive prognostic value of the ferroptosis‐related protein GPX4 and the ferroptosis‐inducing agent SLC7A11 in ovarian cancer. Our clinical analyses revealed that expression of SLC7A11 and GPX4 was higher in the platinum‐resistant group than in the platinum‐sensitive group. Our study also revealed that patients with high co‐expression levels of SLC7A11 and GPX4 had a 60‐fold higher risk of platinum resistance compared with those with low co‐expression. Moreover, we found that patients with a high co‐expression level of SLC7A11 and GPX4 exhibited poorer PFS and OS compared with those with low expression levels of these proteins. Thus, our findings suggest a potential therapeutic target in patients with platinum‐resistant EOC.

### 4.2 Strengths and limitations

Study design is one of the main strengths of our study. Initially, all eligible platinum‐resistant EOC patients were screened out via detailed clinical follow‐up information. Afterwards, we established a platinum‐sensitive group as the control, matched by FIGO stage, age and tumour grade, at a ratio of 1:3. Finally, we employed a random sampling method to select an equal number of patients with three different PFI intervals. Our results from clinical tissue samples were validated using in vitro cytological experiments and database analysis. Another strength of our study lies in the detailed clinical data and follow‐up information of the included patients.

Despite these promising results, some questions remain. One of the limitations of this study is that the number of platinum‐resistant ovarian cancer patients was comparatively small when compared with platinum‐sensitive patients (25.00%, 48/192 cases). Moreover, the role of ferroptosis, SLC7A11 and GPX4 in platinum resistance in EOC patients remains unknown. Further molecular studies are required to understand this association better.

### 4.3 Interpretation of results and implications for clinical practice

Although improved screening, surgery, chemotherapy and targeted therapy have improved survival rates in recent years,^3^, ^24^ effective treatment of patients with platinum‐resistant EOC remains a significant unmet medical need. Hence, it is urgent to explore a novel predictor of platinum resistance and poor prognosis in EOC patients, and develop novel therapeutic target for patients with EOC, especially those with platinum resistance. Most of ovarian cancer patients carry wild‐type *BRCA* with no significant clinical benefits from PARP inhibitors; however, recent studies have shown that this subset of patients is sensitive to targeted‐SLC7A11 ferroptosis inducers.^15^ Based on this knowledge, a potential therapeutic strategy (combination of PARP inhibitor and ferroptosis inducers) for *BRCA*‐proficient ovarian cancer has been proposed.^15^ Recently, it has also been shown that ferroptosis‐related genes such as GPX4 and SLC7A11, could serve as a novel biomarker for predicting the prognosis in breast cancer (another class of *BRCA*‐related cancer) patients.^25^ From this, we conclude that ferroptosis inducers targeting SLC7A11 might be new therapeutic targets for cancer patient with wild‐type *BRCA* by potentiating the efficacy of PARP inhibitors. Thus, we assessed the expression levels of SLC7A11 and GPX4 (a ferroptosis molecular marker) in relation to platinum resistance and prognosis in patients with EOC. We found that targeted‐SLC7A11 ferroptosis strategy may be a potential therapeutic target for EOC patients with platinum resistance.

Most chemotherapeutics, including platinum, can induce oxidative stress and elevate intracellular levels of ROS to kill cancer cells.^26^, ^27^, ^28^ In contrast, ROS‐induced chemoresistance can be triggered by prolonged or excessive oxidative stress.^29^ SLC7A11 is known to maintain intracellular GSH levels and prevent oxidative stress‐induced cell death mechanisms, such as ferroptosis.^9^, ^12^ A high expression level of SLC7A11 was associated with altered cell metabolism, characterised by increased mitochondrial biogenesis, oxidative phosphorylation or ATP production,^30^ leading to increased resistance to cisplatin in cancers.^31^, ^32^ Consistent with previous reports, we found that high SLC7A11 expression was associated with platinum resistance in patients with EOC.

Recently, Lei et al. indicated that SLC7A11 could promote resistance mainly by inhibiting ferroptosis.^14^ There are also reports that platinum‐tolerant cancer cells with FZD7^+^ had altered GSH metabolism, required GPX4 for survival, and were sensitive to eradication via ferroptosis.^33^ In the analysis to determine whether ferroptosis participates in platinum resistance in EOC via SLC7A11, we found a significant positive correlation between SLC7A11 and GPX4 expression in EOC tissues. Thus our study indicates that targeted‐SLC7A11 ferroptosis may act as a potential therapeutic target for platinum resistance in EOC patients.

## 5 CONCLUSIONS

In summary, our study showed that high expression levels of SLC7A11 and GPX4 are associated with platinum resistance in ovarian cancer patients, and that a high co‐expression level of SLC7A11–GPX4 was a superior predictor for poor prognosis and platinum resistance in EOC compared with the corresponding individual parameters (SLC7A11 or GPX4 expression). Moreover, the high co‐expression level of SLC7A11‐GPX4 mayt be a significant independent prognostic factor of poor prognosis and a potential therapeutic target for platinum resistance in patients with ovarian cancer.

## AUTHOR CONTRIBUTIONS

XDW, SZS and JLQ designed the study, performed database analysis, collected the data, performed statistical analyses and drafted the manuscript. WDF participated in the methodological formulation. FYF, JXG, TS and TZ participated in the cytological experiments, data collection and statistical analyses. XDC conceived the study, participated in its design and coordination and revised the final paper. All the authors have read and approved the final version of the article.

## FUNDING INFORMATION

This study was funded by the National Key Technology Research and Development Programme of China in the design of the study (Grant number: 2022YFC2704200; grant recipient: Qinglei Gao), Key Research and Development Program of Zhejiang Province in the collection of data (Grant number: 2019C03010; grant recipient: Xiaodong Cheng), National Science Foundation Committee in the collection and analysis of data for this study (Grant number: 82171939 and 82103505; grant recipients: Jiale Qin and Weidong Fei, respectively), and the Natural Science Foundation of Zhejiang Province in writing the manuscript (Grant number: LQ20H300002; grant recipient: Weidong Fei).

## CONFLICT OF INTERESTS

The authors declare that they have no competing interests.

## ETHICS APPROVAL

This study was approved by the Ethics Committee of Women's Hospital, Zhejiang University School of Medicine (November 2018, IRB‐20200227‐R). The need for informed consent was waived by the IRB, according to the Ethics Committee of Women's Hospital, Zhejiang University School of Medicine.

## CONSENT FOR PUBLICATION

Not applicable.

## Supporting information


Appendix S1

## Data Availability

The datasets generated and/or analysed during the current study are not publicly available due to the use of complicated calculations, but are available from the corresponding author upon reasonable request.
