# Supplementary material for: High co‐expression of SLC7A11 and GPX4 as a predictor of platinum resistance and poor prognosis in patients with epithelial ovarian cancer
Source: BJOG. 2022 Dec 9;129(Suppl 2):40–9. doi: 10.1111/1471-0528.17327 (PMC10108211; doi:10.1111/1471-0528.17327)
Supplement: Supplementary file 1 — Appendix S1 [file BJO-129-40-s001.docx]

**SUPPLEMENTARY MATERIALS**

**MATERIALS AND METHODS**

**Cell culture**

The human ovarian cancer cell lines, A2780 and SKOV3, were obtained from the American Type Culture Collection (ATCC). A2780 and SKOV3 cells were respectively maintained in RPMI 1640 (Gibco, USA) and McCoy 5A (Gibco, USA) medium supplemented with 10% fetal bovine serum (FBS，Gibco, USA) in a 5% CO_2_ atmosphere at 37 °C. The platinum-resistant cell subline, A2780/CisR, was cultured in RPMI 1640 medium supplemented with 10% FBS containing 1 µM cisplatin, whereas SKOV3/CisR cells were cultured in McCoy 5A medium supplemented with 10% FBS containing 1 µM cisplatin. All cells were detached from the culture plates using a 0.25% trypsin/0.02% EDTA solution (Gino Biotech, Shanghai, China). Before A2780/CisR and SKOV3/CisR cells were collected for western blot analyses, cisplatin was removed from the respective medium (the medium containing cisplatin was removed, and fresh medium without cisplatin was added) for 7 days.

**Western blot analysis**

Cells were lysed with RIPA lysis buffer (Beyotime Biotechnology, China) supplemented with PMSF inhibitor (Beyotime Biotechnology, China). Protein lysates (20 μg) were loaded and separated on a 10% SDS-containing polyacrylamide gel (GenScript, China) and then transferred to 0.22-μm polyvinylidene fluoride (PVDF) membranes (Bio-Rad, USA) at 120 V for 60 min. The membranes were blocked with a Tris-buffered saline solution containing Tween-20 (TBST) and 5% nonfat milk for 1 h at room temperature(generally in the range of 23-26℃) and incubated with the primary antibodies overnight at 4 °C. The membranes were then washed twice with TBST for 10 min each time and incubated with the corresponding horseradish peroxidase (HRP)-linked secondary antibodies for 1 h, followed by three washes in TBST for 10 min per wash. The bands were visualized using an enhanced chemiluminescence (ECL) kit (Thermo Fisher Scientific, Waltham, MA, USA) in an ImageQuant LAS400 mini system (GE Healthcare, Germany). Antibodies against the following proteins were used in the experiments: GPX4 (1:1000 in NCM universal antibody diluent, ab125066, Abcam, England), actin (1:5000 in NCM universal antibody diluent, A3854, Sigma, USA), and SLC7A11 (1:1000 in NCM universal antibody diluent, ab37185, Abcam, England), goat anti-rabbit secondary antibody (1:5000 in NCM universal antibody diluent, SA0001-2, Proteintech, USA).

**Cell viability assay**

Cells were seeded into 96-well plates at a density of 5 × 10^3^ cells/well. After one day of growth, the cells were treated with different concentrations of cisplatin (1-48μM) and incubated at 37 °C for 48 h. Cell viability was detected using a CCK-8 assay 48 h after drug treatment. The absorbance at 450 nm was measured using a Varioskan Flash microplate reader (Thermo Fisher Scientific) and the results were calculated using GraphPad Prism software.

**siRNA transfection**

SLC7A11, GPX4, and control siRNA were synthesized by GenePharma (Shanghai, China). Transient transfection was performed using DharmaFECT Transfection Reagents (Thermo Fisher Scientific) following the standard protocol. The target sequences of the siRNAs used in this step are listed in Table S1.

**RNA extraction and RT-qPCR analysis**

Total RNA was extracted using TRIzol reagent (Invitrogen, USA). The extracted RNA was reverse-transcribed using a PrimeScript RT Reagent Kit with a gDNA eraser (Takara, Japan). PCR analysis was conducted using a TB Green Premix Ex Taq (Takara) and a 7900HT Fast Real-Time PCR System (Life Technologies). The primer sequences are listed in Table S2. Relative mRNA expression was calculated using the 2^−ΔΔCt^ method and normalized to β-actin expression.

**GEO database analysis**

The GSE66957 dataset, which contained transcriptome microarray data of 12 normal ovarian and 57 ovarian cancer samples, was downloaded from the GEO database. The expression values of GPX4 and SLC7A11 were extracted, Pearson correlation analysis was performed, and scatter plots were obtained.

**SUPPLEMENTARY TABLES**

**Table S1.** Sequences of siRNAs used in this study and their specific targets.

| Item | Sequence | |
| --- | --- | --- |
| SLC7A11 siRNA-1 | Sense (5′-3′) | GGGUGGAACUCCUCAUAAUTT |
|  | Antisense (5′-3′) | AUUAUGAGGAGUUCCACCCTT |
| SLC7A11 siRNA-2 | Sense (5′-3′) | CCAGGUGGUUUAGAAUAAUTT |
|  | Antisense (5′-3′) | AUUAUUCUAAACCACCUGGTT |
| GPX4 siRNA-1 | Sense (5′-3′) | GGAAGUGGAUGAAGAUCCATT |
|  | Antisense (5′-3′) | UGGAUCUUCAUCCACUUCCTT |
| GPX4 siRNA-2 | Sense (5′-3′) | GGAGUAACGAAGAGAUCAATT |
|  | Antisense (5′-3′) | UUGAUCUCUUCGUUACUCCTT |
| Ctrl siRNA | Sense (5′-3′) | UUCUCCGAACGUGUCACGUTT |
|  | Antisense (5′-3′) | ACGUGACACGUUCGGAGAATT |

**Table S2.** Sequences of primers used for qRT-PCR in this study.

| Item | Sequence | |
| --- | --- | --- |
| β-actin | Forward (5′-3′) | CATGTACGTTGCTATCCAGGC |
|  | Reverse (5′-3′) | CTCCTTAATGTCACGCACGAT |
| SLC7A11 | Forward (5′-3′) | TCTCCAAAGGAGGTTACCTGC |
|  | Reverse (5′-3′) | AGACTCCCCTCAGTAAAGTGAC |
| GPX4 | Forward (5′-3′) | GCCTTCCCGTGTAACCAGT |
|  | Reverse (5′-3′) | GCGAACTCTTTGATCTCTTCGT |

**Table S3.** Differential expression analysis of SLC7A11 in all four groups.

| Group | SLC7A11-low expression  No. (%) | SLC7A11-high expression  No. (%) | *P*-value |
| --- | --- | --- | --- |
| Number of patients | 149 (77.60) | 43 (22.40) | <0.001*** |
| 1 | 18 (37.50) | 30 (62.50) |  |
| 2 | 34 (80.95) | 8 (19.05) |  |
| 3 | 51 (98.08) | 1 (1.92) |  |
| 4 | 46 (92.00) | 4 (8.00) |  |

Categorical data are presented as absolute values (%). *P*-values were calculated using the chi-squared test.

**Table S4.** Pairwise comparison of the differential expression of SLC7A11 in each group.

| Group (I vs. J) | *P*-value |
| --- | --- |
| 1 vs. 2 | < 0.001* |
| 1 vs. 3 | < 0.001* |
| 1 vs. 4 | < 0.001* |
| 2 vs. 3 | 0.01† |
| 2 vs. 4 | 0.117 |
| 3 vs. 4 | 0.200† |

*P*-values were calculated using the chi-squared test; †these values were calculated using Fisher’s exact tests since more than 20% of the cells had expected frequencies < 5. *adjusted *P*-values < 0.0083(0.05/6)

**Table S5.** Differential expression analysis of GPX4 in all four groups

| Group | GPX4-low expression  No. (%) | GPX4-high expression  No. (%) | *P*-value |
| --- | --- | --- | --- |
| Number of patients | 148 (77.08) | 44 (22.92) | < 0.001*** |
| 1 | 15 (31.25) | 33 (68.75) |  |
| 2 | 33 (78.57) | 9 (21.43) |  |
| 3 | 52 (100.00) | 0 (0.00) |  |
| 4 | 48 (96.00) | 2 (4.00) |  |

Categorical data are presented as absolute values (%). *P*-values were calculated using the chi-squared test.

**Table S6.** Differential expression analysis of GPX4 from other groups.

| Group (I vs. J) | *P*-value |
| --- | --- |
| 1 vs. 2 | < 0.001* |
| 1 vs. 3 | < 0.001* |
| 1 vs. 4 | < 0.001* |
| 2 vs. 3 | < 0.001*† |
| 2 vs. 4 | 0.02 |
| 3 vs. 4 | 0.238 |

*P*-values were calculated using the chi-squared test; †these values were calculated using Fisher’s exact tests since more than 20% of the cells had expected frequencies < 5. *adjusted *P-*values < 0.0083 (0.05/6).

**Table S7.** Logistics regression analysis of the association of SLC7A11 and GPX4 with platinum resistance.

| Factors | Waldχ^2^ | OR | *P*-value |
| --- | --- | --- | --- |
| SLC7A11 | 11.759 | 1.588 | 0.001** |
| GPX4 | 26.219 | 1.931 | <0.001*** |

***P* < 0.01; ****P* < 0.001.

**Table S8.** Univariate Cox analysis for overall survival (OS) and progression-free survival (PFS) associated with SLC7A11, GPX4, and SLC7A11-GPX4 expression.

|  |  | PFS |  |  | OS |  |
| --- | --- | --- | --- | --- | --- | --- |
|  | HR | 95%CI | *P*-value | HR | 95%CI | *P*-value |
| SLC7A11 | 3.219 | 2.203-4.705 | < 0.001*** | 4.426 | 2.833-6.914 | < 0.001*** |
| GPX4 | 4.545 | 3.103-6.656 | < 0.001*** | 4.935 | 3.196-7.620 | < 0.001*** |
| SLC7A11-GPX4  GPX4 4GPX4 | 6.991 | 4.750-10.290 | < 0.001*** | 5.884 | 3.821-9.063 | < 0.001*** |

****P* < 0.001.

**Table S9.** Multivariate Cox proportional hazards regression analysis for PFS.

|  | β | Waldχ^2^ | Exp(β) | 95% CI | *P*-value |
| --- | --- | --- | --- | --- | --- |
| Age (years) | 0.493 | 3.604 | 1.637 | 0.984-2.722 | 0.058 |
| FIGO stage | -0.104 | 0.195 | 0.902 | 0.569-1.428 | 0.659 |
| Ascitic fluid volume (mL) | 0.333 | 2.737 | 1.395 | 0.940-2.069 | 0.098 |
| Serum CA125 (U/mL) | 0.295 | 2.120 | 1.343 | 0.903-1.997 | 0.145 |
| Primary surgery | 0.411 | 3.723 | 1.508 | 0.994-2.290 | 0.054 |
| Menopause status | -0.530 | 4.460 | 0.589 | 0.360-0.963 | 0.035* |
| Tumor grade | 0.298 | 0.775 | 1.347 | 0.694-2.615 | 0.379 |
| GPX4 expression | 1.008 | 17.984 | 2.740 | 1.720-4.366 | < 0.001*** |
| SLC7A11 expression | 0.855 | 14.328 | 2.351 | 1.510-3.659 | < 0.001*** |

**P* < 0.05; ***P* < 0.01; ****P* < 0.001.

**Table S10**. Multivariate Cox proportional hazards regression analysis for OS

|  | β | Waldχ^2^ | Exp(β) | 95% CI | *P*-value |
| --- | --- | --- | --- | --- | --- |
| Age(years) | 0.228 | 0.462 | 1.257 | 0.650-2.427 | 0.497 |
| FIGO stage | -0.231 | 0.562 | 0.794 | 0.434-1.451 | 0.453 |
| Ascitic fluid volume (mL) | 0.538 | 4.479 | 1.712 | 1.041-2.816 | 0.034* |
| Serum CA125 (U/mL) | 0.237 | 0.884 | 1.267 | 0.774-2.075 | 0.347 |
| Primary surgery | 0.458 | 3.532 | 1.581 | 0.981-2.550 | 0.060 |
| Menopause status | -0.098 | 0.090 | 0.907 | 0.479-1.717 | 0.764 |
| Tumor grade | 0.471 | 0.981 | 1.601 | 0.631-4.063 | 0.322 |
| GPX4 expression | 0.814 | 8.655 | 2.258 | 1.312-3.885 | 0.003** |
| SLC7A11 expression | 1.048 | 14.359 | 2.851 | 1.658-4.902 | < 0.001*** |

**P* < 0.05; ***P* < 0.01; ****P* < 0.001.

**Table S11.** Results of the multivariate Cox analysis for PFS with SLC7A11-GPX4 expression.

|  | β | Waldχ^2^ | Exp(β) | 95% CI | *P*-value |
| --- | --- | --- | --- | --- | --- |
| Age (years) | 0.410 | 2.265 | 1.507 | 0.883-2.572 | 0.132 |
| FIGO stage | -0.036 | 0.024 | 0.964 | 0.610-1.523 | 0.876 |
| Ascitic fluid volume (mL) | 0.117 | 0.334 | 1.124 | 0.756-1.673 | 0.563 |
| Serum CA125 (U/mL) | 0.303 | 2.263 | 1.354 | 0.912-2.010 | 0.133 |
| Primary surgery | 0.401 | 3.522 | 1.493 | 0.982-2.268 | 0.061 |
| Menopause status | -0.409 | 2.330 | 0.664 | 0.393-1.123 | 0.127 |
| Tumor grade | 0.341 | 1.020 | 1.406 | 0.726-2.724 | 0.312 |
| SLC7A11-GPX4 | 1.745 | 65.839 | 5.729 | 3.758-8.733 | < 0.001*** |

****P* < 0.001.

**Table S12** Multivariate Cox analysis results for OS with SLC7A11-GPX4 expression.

|  | β | Waldχ^2^ | Exp(β) | 95% CI | *P*-value |
| --- | --- | --- | --- | --- | --- |
| Age (years) | 0.138 | 0.158 | 1.148 | 0.581-2.271 | 0.691 |
| FIGO stage | -0.130 | 0.183 | 0.878 | 0.484-1.592 | 0.669 |
| Ascitic fluid volume (mL) | 0.323 | 1.648 | 1.381 | 0.844-2.262 | 0.199 |
| Serum CA125 (U/mL) | 0.246 | 0.973 | 1.279 | 0.785-2.084 | 0.324 |
| Primary surgery | 0.384 | 2.383 | 1.469 | 0.902-2.392 | 0.123 |
| Menopause status | -0.050 | 0.022 | 0.951 | 0.489-1.849 | 0.882 |
| Tumor grade | 0.290 | 0.374 | 1.336 | 0.528-3.380 | 0.541 |
| SLC7A11-GPX4 | 1.491 | 38.005 | 4.442 | 2.765-7.136 | < 0.001*** |

****P* < 0.001.

**SUPPLEMENTARY FIGURES**


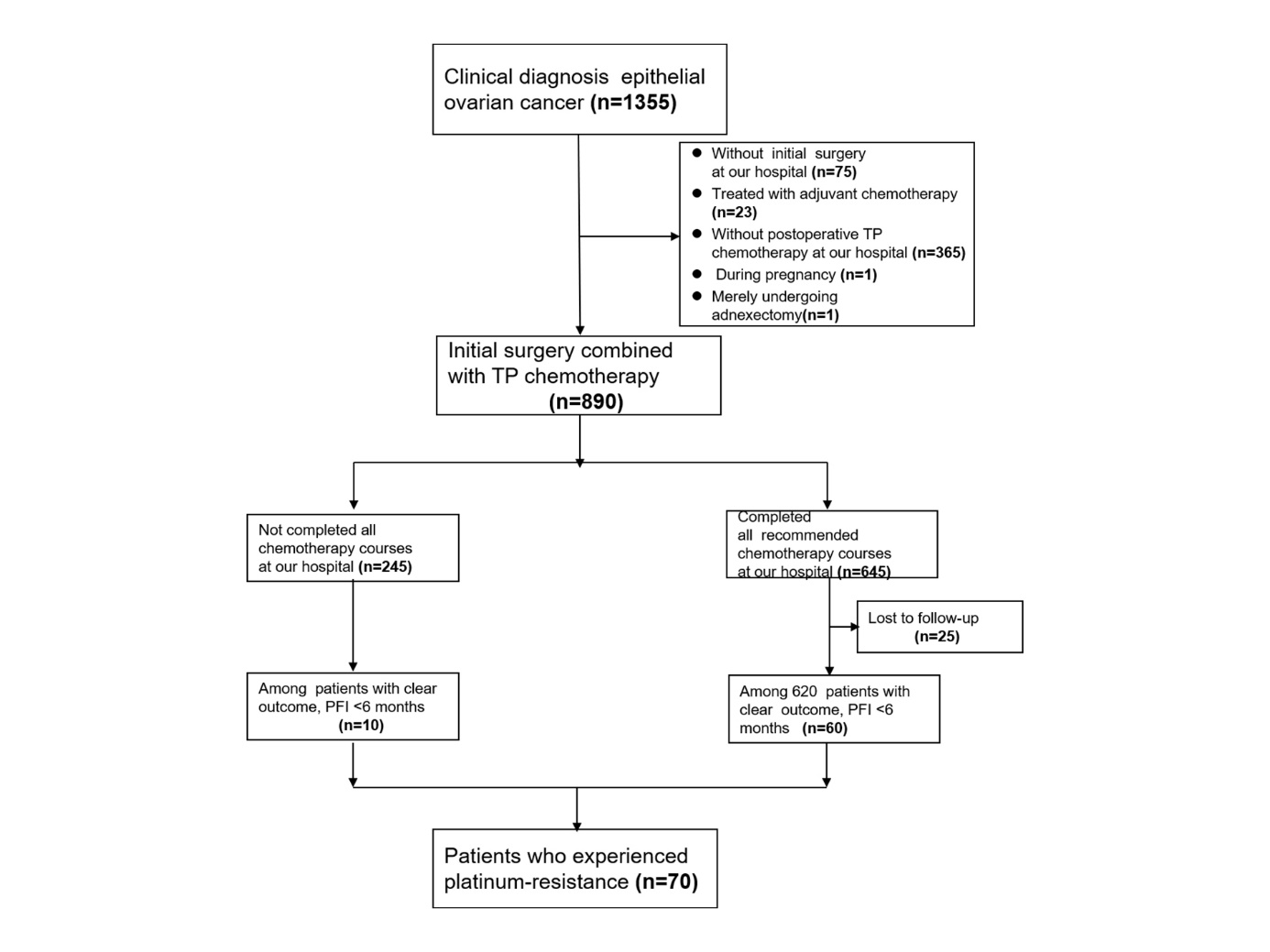


**Figure S1. The enrollment flowchart for the platinum-resistant subjects.**


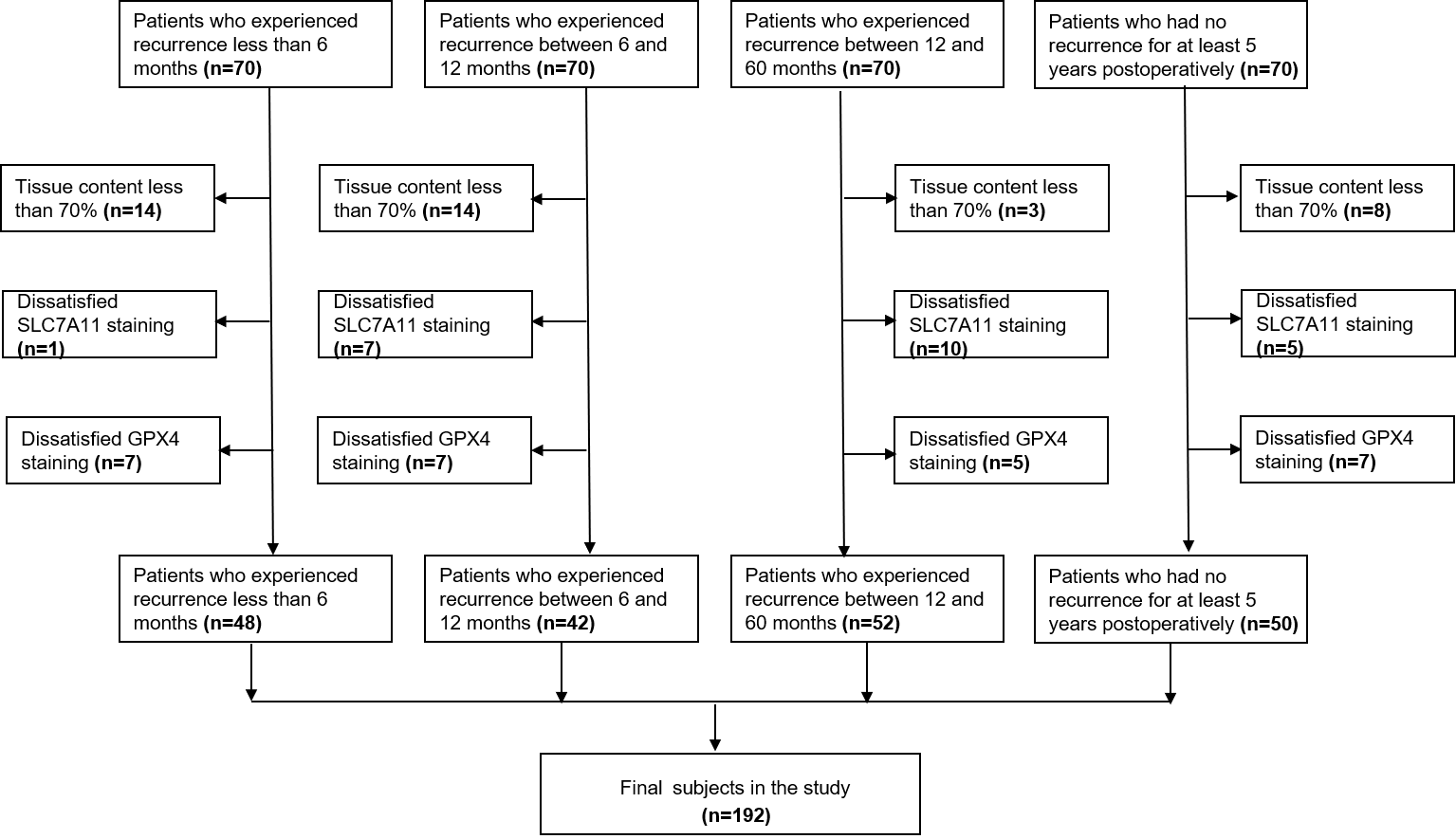


**Figure S2. Enrollment flow chart for the study subjects.**


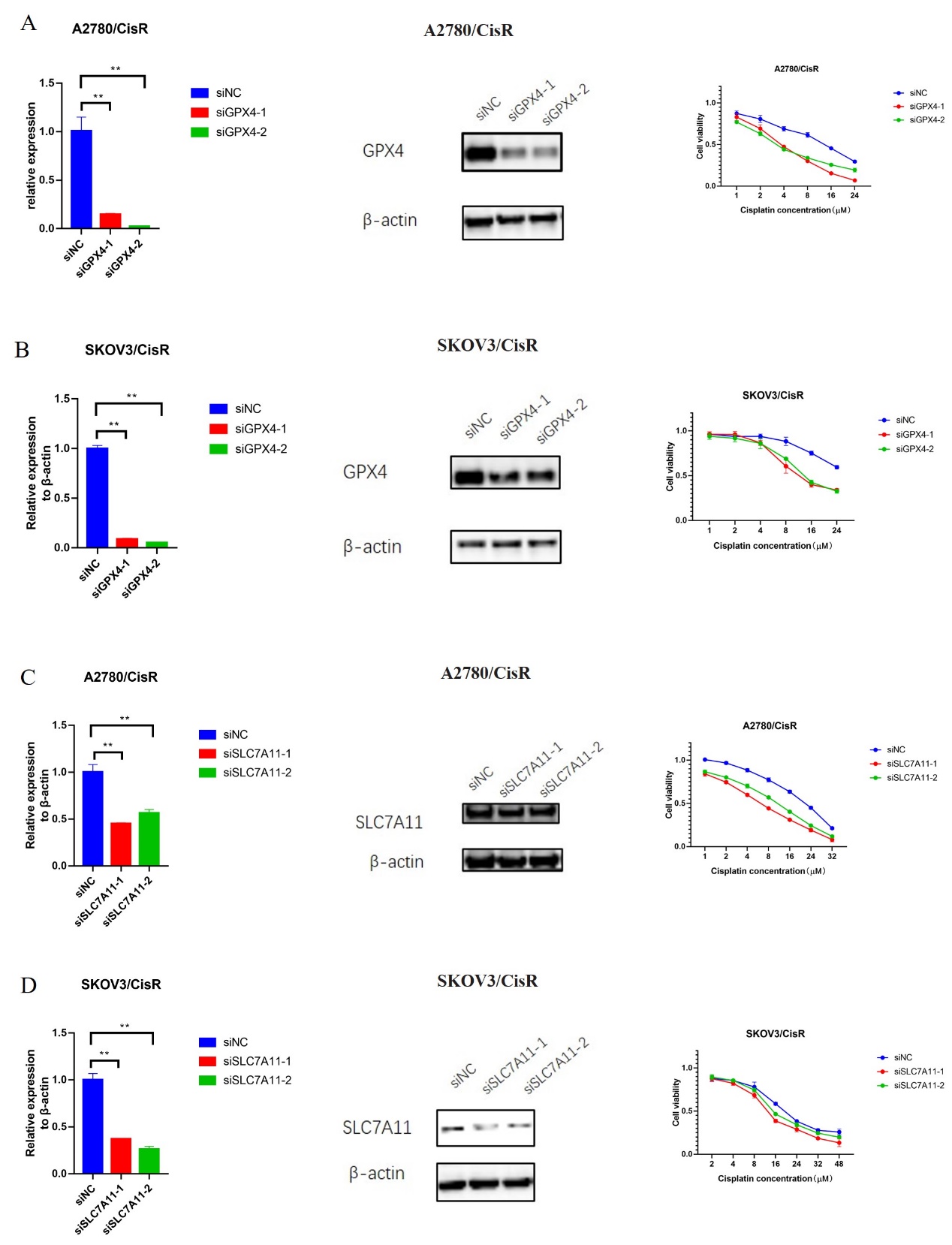


**Figure S3. Inhibition of SLC7A11 and GPX4 expression decreased platinum resistance in ovarian cancer cells. (A-B)** A2780/CisR and SKOV3/CisR cells transfected with GPX4 siRNA. **(C-D)** A2780/CisR and SKOV3/CisR cells transfected with SLC7A11 siRNA. After transfection, the transcriptional levels (as determined using RT-PCR, leftmost column) of GPX4 and SLC7A11 and protein expressions (as determined using western blotting analysis, middle column) of GPX4 and SLC7A11 decreased. After transfection, siRNA-induced SLC7A11 and GPX4 inhibition caused a dramatic decrease in platinum-resistant cell viability as determined through the CCK8 assay (rightmost column).


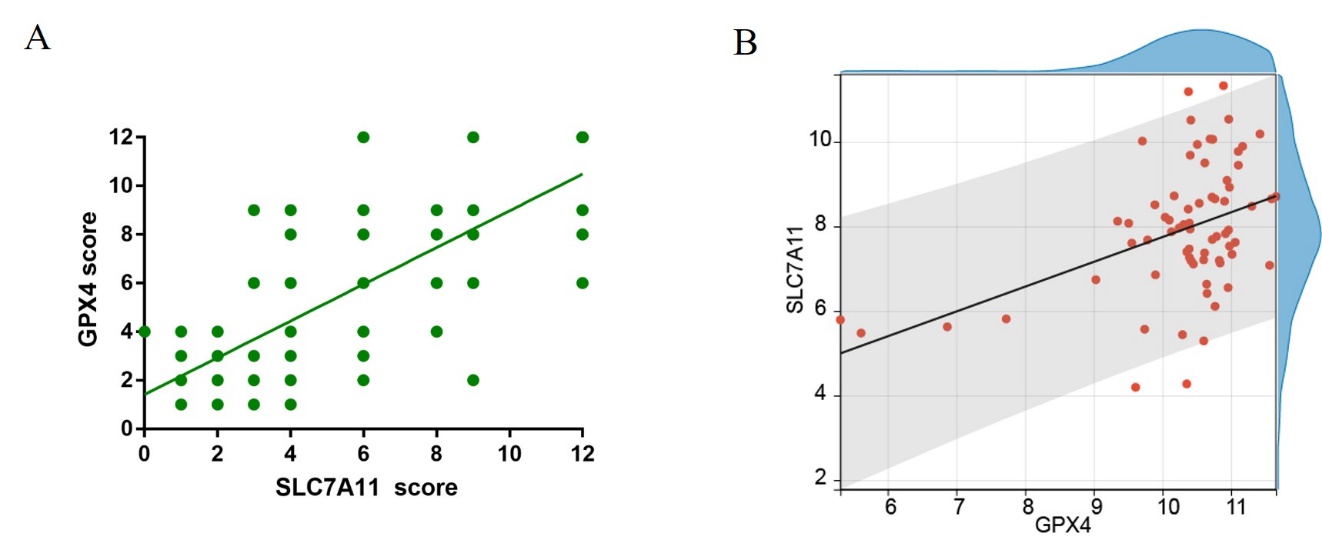


**Figure S4. Correlation between the expression of SLC7A11 and GPX4.** **(A)** The protein expression levels of SLC7A11 and GPX4 were positively correlated in EOC tissues of our included 192 patients (r = 0.677, Pearson correlation). **(B)** The mRNA expression levels of SLC7A11 and GPX4 were positively correlated in the 12 normal ovarian and 57 ovarian cancer samples from the GSE66957 dataset downloaded from the GEO database (r = 0.43, Pearson correlation).


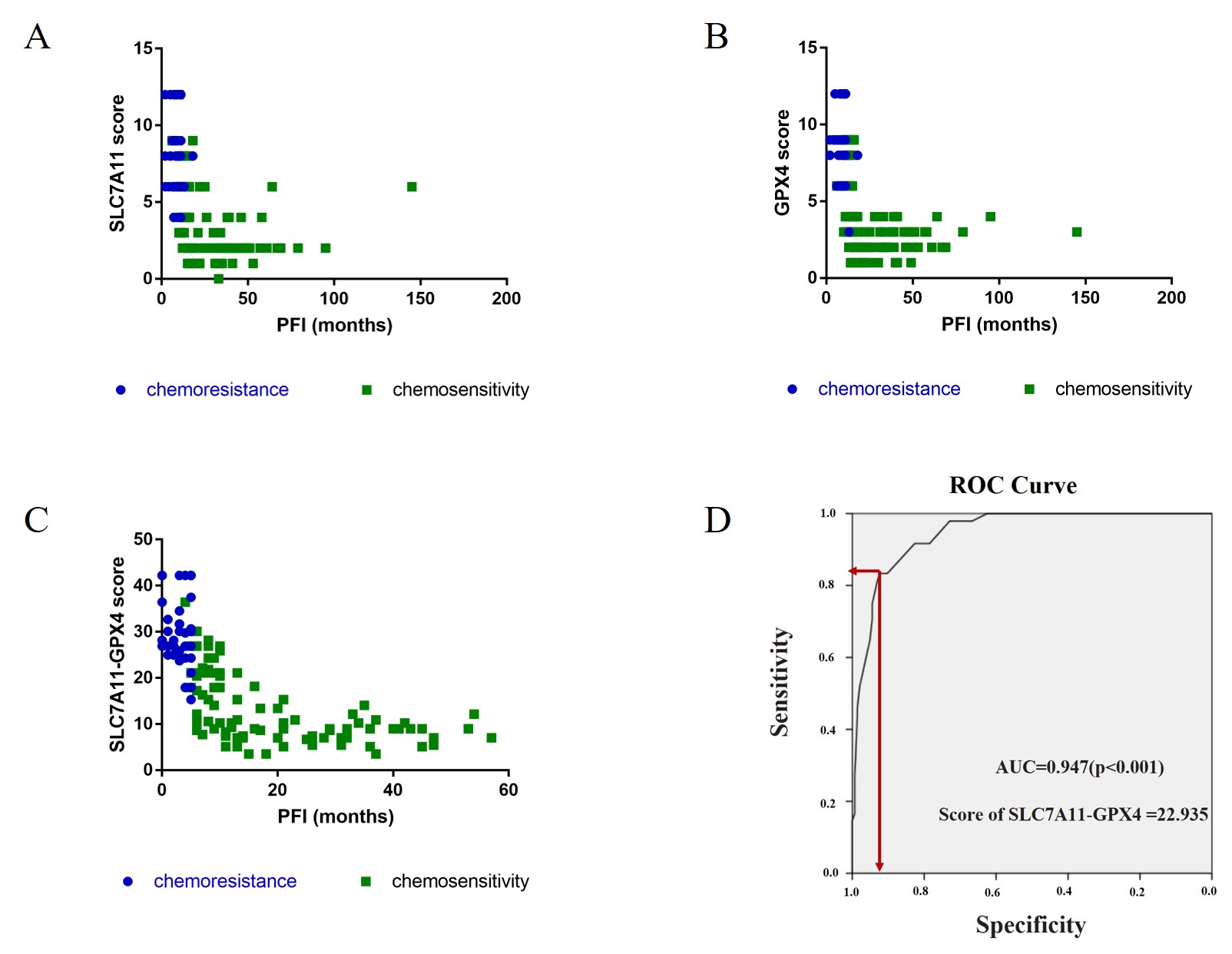
**Figure S5. Association of SLC7A11 and GPX4 expression with platinum resistance. (A)** Scatter plot of the SLC7A11 immunohistochemical score in EOC patients with different PFI. **(B)** Scatter plot of the GPX4 immunohistochemical score in EOC patients with different PFI. **(C)** Scatter plot of the SLC7A11-GPX4 score in EOC patients with different PFI. **(D)** ROC curve of the SLC7A11-GPX4 score as a predictor for platinum resistance.
